# Supplementary material for: Performance of Classification Models of Toxins Based on Raman Spectroscopy Using Machine Learning Algorithms
Source: Molecules. 2023 Dec 29;29(1):197. doi: 10.3390/molecules29010197 (PMC10780255; doi:10.3390/molecules29010197)
Supplement: Supplementary file 1 [file molecules-29-00197-s001.zip › molecules-2728211-supplementary.pdf]

Performance of Classification Models of Toxins Based on Raman Spectroscopy Using Machine Learning Algorithms

Pengjie Zhang, Bing Liu, Xihui Mu, Jiwei Xu, Bin Du, Jiang Wang, Zhiwei Liu and Zhaoyang Tong *

State Key Laboratory of NBC Protection for Civilian, Beijing 102205, China; zpjbit@163.com (P.Z.); lbfhyjy@sohu.com (B.L.); mxh0511@sohu.com (X.M.); xujw14@mail.ustc.edu.cn (J.X.); dubin51979@163.com (B.D.); roverman@163.com (J.W.); liuzhw07@lzu.edu.cn (Z.L.)

* Correspondence: billzytong@126.com

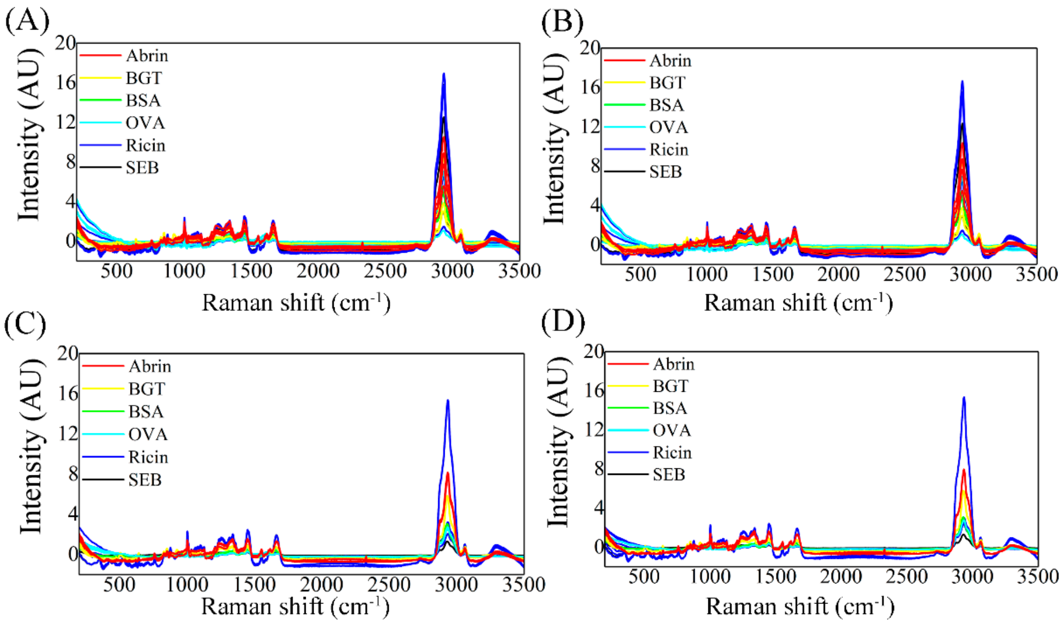

Figure S1. Original region spectra of samples processed by raw (A), WT (B), MSC (C), and MSC-SG (D).

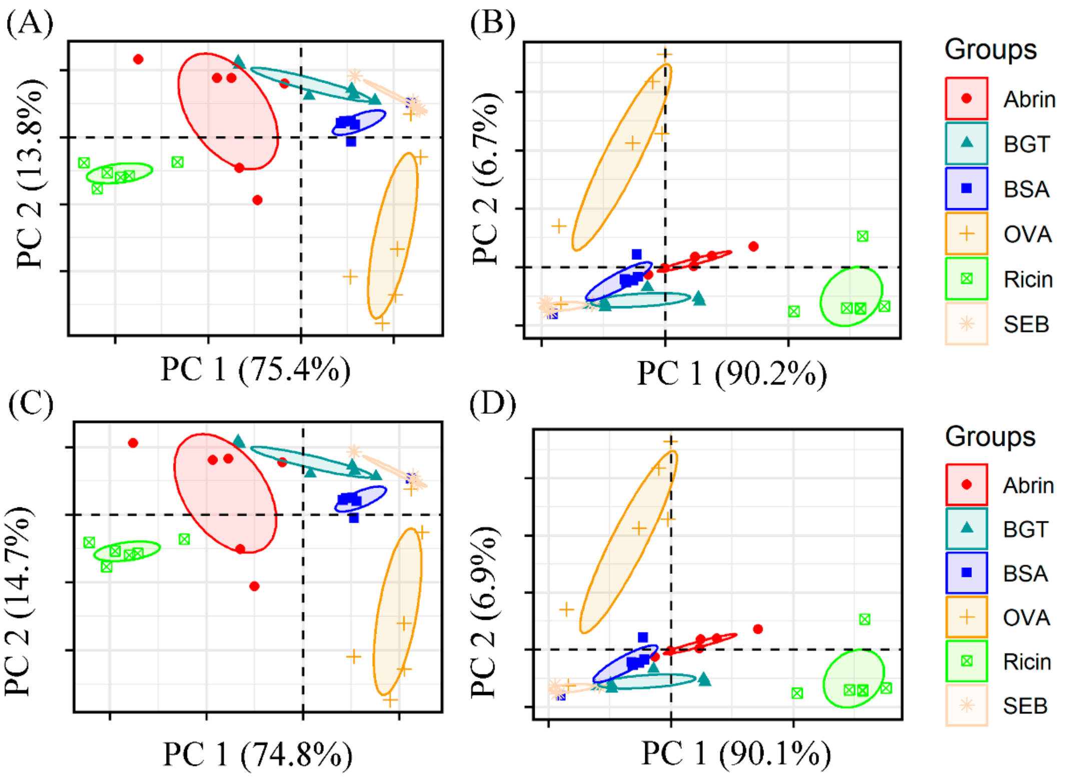

Figure S2. PCA score plots of fingerprint region (left) and original region spectra (right) with different pretreatments through Raw (A/ B) and WT (C/D).

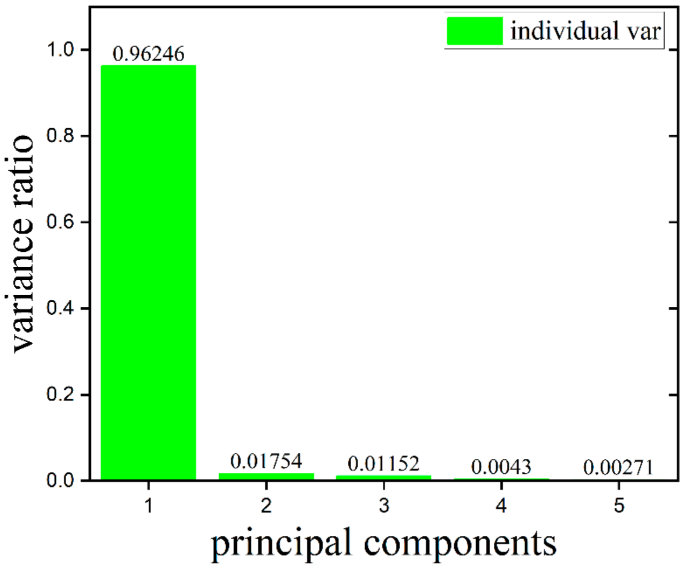

Figure S3. Principal component (PC) variance contribution rate of OS-MSC-SG data.

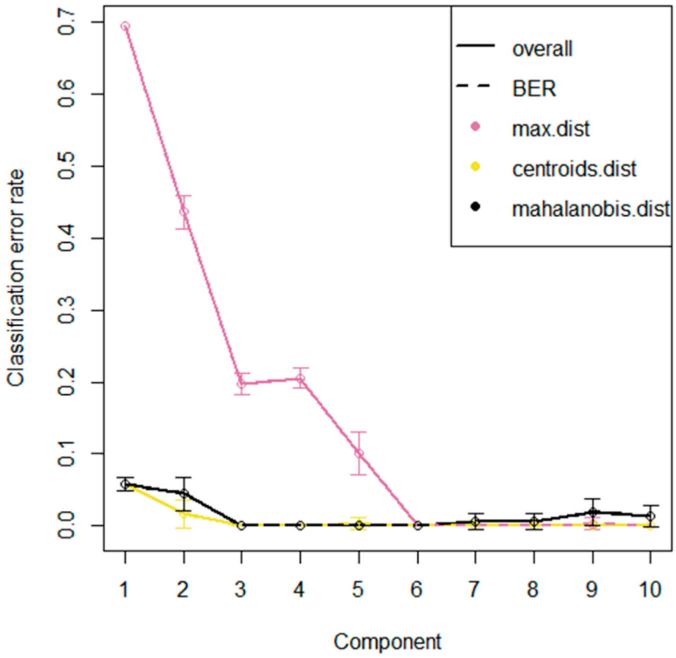

Figure S4. The classification error rate of PLS-DA.

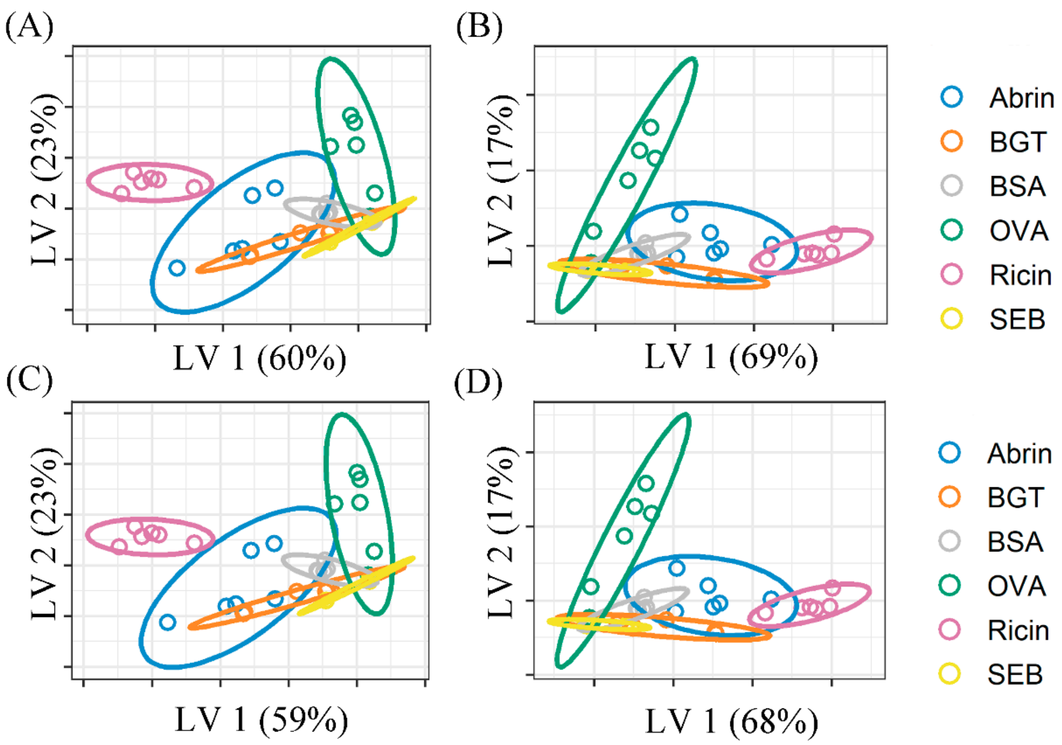

Figure S5. PLS-DA score plots of fingerprint region (left) and original region spectra (right) with different pretreatments through Raw (A/ B) and WT (C/D).

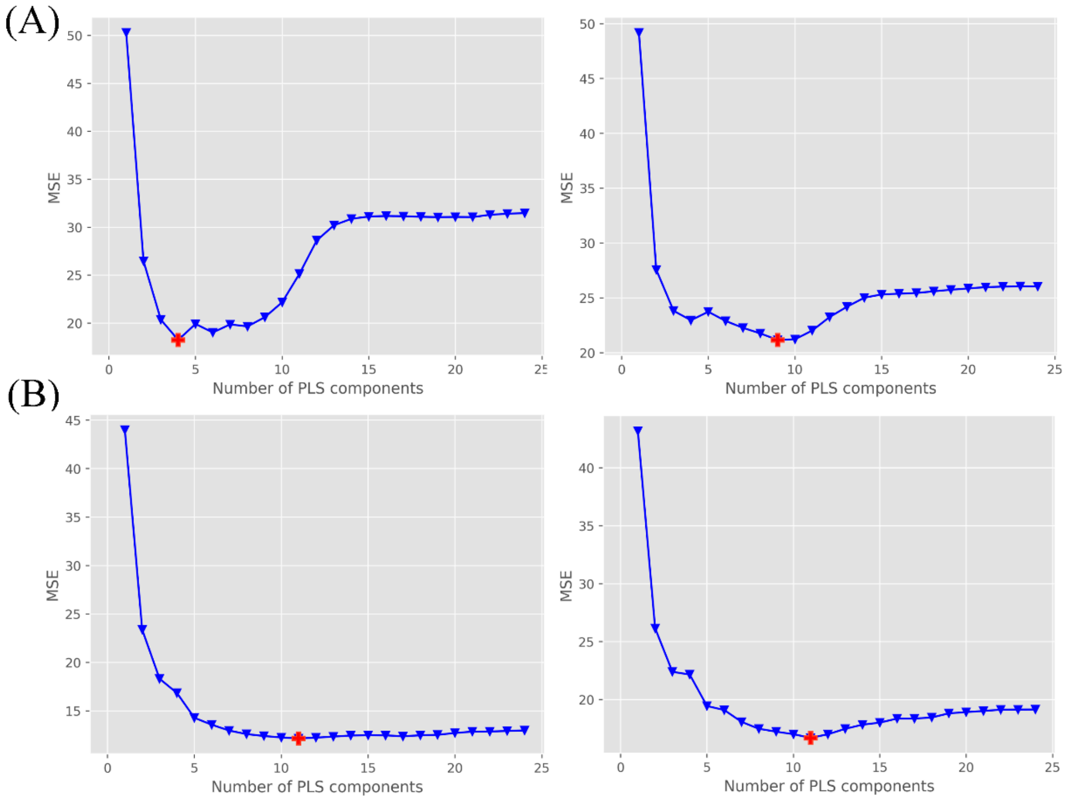

Figure S6. The suggested number of PLS components raw of (A) and MSC-SG (B) spectra. (Figure left: fingerprint Raman region, figure right: original range, respectively).

Table S1. The confusion matrices of the K-means for Raman spectra and PC data. The data were processed by WT method. The classification results of K-means are expressed in percentage.

Methods	Class	Spectra data						PCs data					
		*Abrin	*BGT	*BSA	*OVA	*Ricin	*SEB	*Abrin	*BGT	*BSA	*OVA	*Ricin	*SEB
raw	Abrin	66.7		16.3				66.7		16.3			
	BGT	33.3	50	16.7					0				
	BSA			83.3			16.7			83.3			16.7
	OVA				66.7		16.3		33.3		50		16.7
	Ricin					100		16.7				83.3	
	SEB						100						100
WT	Abrin	66.7		16.3				83.3		16.7			
	BGT	33.3	50	16.7					0				
	BSA			83.3			16.7	16.7		83.3			
	OVA				66.7		16.3	16.7	33.3		33.3		16.7
	Ricin					100		16.7				83.3	
	SEB						100						0

*Abrin: The predicted class of Abrin. The red number indicates that the sample has not been fully classified correctly, while the green number indicates that the sample has been completely and correctly classified.

Table S2. Categories prediction of average spectral data of K-means model.

Method	Class	*Abrin	*BGT	*BSA	*Ricin	*OVA	*SEB
Raw	Abrin	100					
	BGT		100				
	BSA			100			
	OVA				100		
	Ricin					100	
	SEB						100
MSC	Abrin	100					
	BGT		100				
	BSA			100			
	OVA				100		
	Ricin					100	
	SEB						100
MSC-SG	Abrin	100					
	BGT		100				
	BSA			100			
	OVA				100		
	Ricin					100	
	SEB						100
WT	Abrin	100					
	BGT		100				
	BSA			100			
	OVA				100		
	Ricin					100	
	SEB						100

Table S3. The confusion matrix for PLS-DA classification model.

samples	AUC	p-value
Abrin	1	0.0001333
BGT	1	0.0001333
BSA	1	0.0001333
OVA	1	0.0001333
Ricin	1	0.0001333
SEB	1	0.0001333

Table S4. The running time of the programs.

Program	Time (s)
PCA	30.63
K-means	5.29
PLS-DA	15.80
PLS	12.27

* s represents seconds.
